# Targeted NGF siRNA Delivery Attenuates Sympathetic Nerve Sprouting and Deteriorates Cardiac Dysfunction in Rats with Myocardial Infarction

**DOI:** 10.1371/journal.pone.0095106

**Published:** 2014-04-22

**Authors:** Hesheng Hu, Yongli Xuan, Ye Wang, Mei Xue, Fei Suo, Xiaolu Li, Wenjuan Cheng, Xinran Li, Jie Yin, Ju Liu, Suhua Yan

**Affiliations:** 1 School of Medicine, Shandong University, Jinan, China; 2 Department of Cardiology, Shandong Provincial Qianfoshan Hospital, Shandong University, Jinan, China; 3 Medical Research Center, Shandong Provincial Qianfoshan Hospital, Shandong University, Jinan, China; UAE University, Faculty of Medicine & Health Sciences, United Arab Emirates

## Abstract

Nerve growth factor (NGF) is involved in nerve sprouting, hyper-innervation, angiogenesis, anti-apoptosis, and preservation of cardiac function after myocardial infarction (MI). Positively modulating NGF expression may represent a novel pharmacological strategy to improve post-infarction prognosis. In this study, lentivirus encoding NGF short interfering RNA (siRNA) was prepared, and MI was modeled in the rat using left anterior descending coronary artery ligation. Rats were randomly grouped to receive intramyocardial injection of lentiviral solution containing NGF-siRNA (n = 19, MI-SiNGF group), lentiviral solution containing empty vector (n = 18, MI-GFP group) or 0.9% NaCl solution (n = 18, MI-control group), or to receive thoracotomy and pericardiotomy (n = 17, sham-operated group). At 1, 2, 4, and 8 wk after transduction, rats in the MI-control group had higher levels of NGF mRNA and protein than those in the sham-operated group, rats in the MI-GFP group showed similar levels as the MI-control group, and rats in the MI-SiNGF group had lower levels compared to the MI-GFP group, indicating that MI model was successfully established and NGF siRNA effectively inhibited the expression of NGF. At 8 wk, echocardiographic and hemodynamic studies revealed a more severe cardiac dysfunction in the MI-siRNA group compared to the MI-GFP group. Moreover, rats in the MI-siRNA group had lower mRNA and protein expression levels of tyrosine hydroxylase (TH) and growth-associated protein 43-positive nerve fibers (GAP-43) at both the infarcted border and within the non-infarcted left ventricles (LV). NGF silencing also reduced the vascular endothelial growth factor (VEGF) expression and decreased the arteriolar and capillary densities at the infarcted border compared to the MI-GFP group. Histological analysis indicated a large infarcted size in the MI-SiNGF group. These findings suggested that endogenous NGF silencing attenuated sympathetic nerve sprouting and angiogenesis, enlarged the infarct size, aggravated cardiac dysfunction, and potentially contributed to an unfavorable prognosis after MI.

## Introduction

Myocardial infarction (MI) is a major cause of death and disability worldwide [1]. Advances in medicine have markedly improved the long-term survival and quality of life in MI patients [2]. However, such clinical innovations can halt, but not reverse the underlying disease process. The prevalence of arrhythmias and heart failure remain unacceptably high in post-infarction patients [3], [4]. Those post-MI complications are correlated with anatomical (myocardial, vascular, neural) and cardiac electrical remodeling initiated by the infarction. Activation of neurohormonal factors by MI has been proven to be critical to this remodeling [5], [6]. Among these factors, nerve growth factor (NGF) is recognized to play an important role in post-infarction remodeling. As a member of the neurotrophin family, NGF is critical for the differentiation, survival, and synaptic activity of sympathetic nerves during human development and after cardiac injury [7], [8]. NGF is rapidly and persistently up-regulated to trigger sympathetic nerve sprouting in infarcted hearts [9], [10]. Both endogenous and ectogenic up-regulation of NGF after MI can promote sympathetic nerve sprouting and hyper-innervation (neural remodeling) which contribute to arrhythmogenesis and sudden cardiac death [9]–[12]. Furthermore, several publications described that drug interventions improve sympathetic nerve sprouting and inhibite hyper-innervation partly by down-regulating myocardial NGF expression in infarcted hearts [13]–[16]. These findings indicate that modulation of NGF expression could regulate sympathetic innervation patterns, providing potential access points for novel therapeutic strategies to prevent lethal arrhythmias and sudden cardiac death.

Moreover, NGF has also been shown to have essential cardiovascular protective functions that are independent of neural regulation of cardiac function [17], [18]. NGF can promote angiogenesis and cardiomyocyte survival [19]. Specifically, NGF gene transfer in a murine model ameliorated cardiomyocyte survival, promoted neovascularization, and improved myocardial blood flow and cardiac function in infarcted mouse hearts [20]. Collectively, up-regulation of NGF expression in post- infarcted hearts exerts both harmful (proarrhythmia) and protective effects (myocardial and vascular repair). These data underscore the need for further studies to elucidate the complicated picture of NGF in post-infarcted hearts. Whether modulating NGF via molecular approach could contribute to counteract some of the negative effects associated with NGF decline or not is still unknown.

In the present study, we firstly reported that NGF was down-regulated via direct intramyocardial administration of a lentiviral NGF short interfering RNA (siRNA) vector in coronary artery-ligated rats. Sympathetic nerve sprouting and density, infarct size, capillary and arteriolar density, and cardiac function were evaluated to elucidate cardiac pathophysiological processes and assess the prognosis.

## Materials and Methods

Male Wistar rats (eight weeks-of-age, 280–300 g) were purchased from animal center of Shandong University. Animals were housed under 12 h/12 h light/dark cycle in a temperature-controlled room (23°C), with access to food and water ad libitum. All procedures involving animals were conducted in accordance with the Guide for the Care and Use of Laboratory Animals (NIH, 1996, No.85–23) and were approved by the Animal Care Committee of Shandong University.

### Preparation of Lentiviral-NGF-siRNA Vector

SiRNA sequences targeting rat NGF (GenBank Acc. No. J04154.1) were designed using a siRNA design tool (Ambion, Austin, TX, USA) and screened according to the guidelines reported by Elbashir SM *et al*. [21]. Three selected siRNA targets with short hairpin frame were synthesized, and the corresponding DNA oligonucleotides (oligo) were cloned into a lentiviral expression vector. All constructs were verified by DNA sequencing. The verified plasmids were then transfected into myocardial cells to select the most effective siRNA target as described previously [22]. As a result, the optimal target (sequence: 5′-CAGGACTTAAAGGCTCTTAAT-3′) was selected. And the lentivirus expressing the optimal siRNA targeting NGF was propagated and harvested using a virus-packaging system (TELEBIO, Shanghai, China). Viral titers were then measured. Both the lentivirus-NGF-siRNA and negative control vectors contained the sequences encoding green fluorescent protein (GFP). The siRNA construct (GFPi) targeting the reporter gene eGFP was included as control.

### MI Model and NGF siRNA Transduction *in vivo*

In this study, 95 male Wistar rats were randomized into treatment and sham-operated groups. Each rat was anesthetized with 3% sodium pentobarbital (ip, 30 mg/kg), intubated via tracheotomy, and ventilated with a small-animal ventilator (HX-300S, TME, Chengdu, China). The heart was exposed through a fourth intracostal left lateral thoracotomy, and MI was modeled by permanently ligating the left anterior descending (LAD) artery approximately 2 mm from its origin as previously described [14], [15]. Thirty minutes after ligation, the survived rats with MI were randomly divided into MI-SiNGF (n = 19, in which rats were injected with virus solution including NGF-siRNA, 1.09×10^9 ^TU/ml), MI-GFP (n = 18, in which rats were injected with negative control virus solution), and MI-control groups (n = 18, in which rats were injected with 0.9% NaCl solution). Each treatment solution (80 µl) was intramyocardially injected at four sites at the infarcted border (∼2 mm around the infarcted area) of the left ventricle at a depth of 1–2 mm with a 30-gauge needle as previously reported [23]. Rats in the sham-operated group (n = 17) only underwent thoracotomy and pericardiotomy. After incision was closed, the rats were allowed to recover from anesthesia in a heated box and then returned to their cages.

### Echocardiographic and Hemodynamic Study

Eight weeks after transduction, animals were sedated. An echocardiographic system equipped with a 14-MHz linear transducer was used to record the M-mode echocardiogram of the left ventricle (LV). The measurement was performed in a blinded manner by an echocardiographic expert. Animals were then intubated and mechanically ventilated. For hemodynamic analysis, a 2F microtip pressure-volume catheter (SPR-869, Millar, Houston, TX, USA) was used to estimate left ventricular pressure and volume. The volume was calibrated by the method of relative volume unit (RVU) calibration as previously reported [24]. Briefly, a volume calibration cuvette with known diameter (AD Instruments, Sydney, Australia) was placed into a 37°C prewarmed water bath and rapidly filled the first 5 holes with fresh heparinized, warm blood. Then the catheter electrode was submerged into the blood-filled cuvette and the conductance was recorded. After performing calibration procedure, the catheter was inserted into the right carotid artery to measure mean arterial blood pressure (MAP) and heart rate (HR). Then, the transducer was advanced from the right carotid artery into the LV to measure LV end-systolic pressure (LVESP) and LV end-diastolic pressure (LVEDP). Then, the maximal rates of increase and decrease in LV pressure (dP/dt_max_ and dP/dt_min_), end-diastolic volume (EDV), and end-systolic volume (ESV) were obtained using LabChart Pro software (AD Instruments, Sydney, Australia). After the hemodynamic study, cardiac tissue was sampled and processed using the methods that were described below.

### Immunohistochemistry (IHC) and Masson’s Trichrome Staining

Expressions of tyrosine hydroxylase (TH) and growth-associated protein 43 (GAP-43) were examined by IHC staining using mouse anti-TH (1∶500; Millipore, Billerica, MA, USA) and rabbit anti-GAP-43 (1∶100; Abcam, Cambridge, UK) as primary antibodies. Anti-CD31 (1∶100; Santa Cruz, CA, USA) and anti-alpha-smooth muscle actin (α-SMA, 1∶100; Boster, Wuhan, China) were used to identify capillaries [25] and arterioles, respectively.

Paraffin sections were deparaffinized, rehydrated, and incubated in 3% hydrogen peroxide solution to inactivate endogenous peroxidase. Slides were then treated with 0.1 M citric acid buffer for 15 min at 92–98°C in a microwave oven and cooled at room temperature. After incubated with serum-free protein blocking buffer (ZSGB-BIO, Beijing, China), sections were incubated with primary antibodies overnight at 4°C, rinsed and incubated in horseradish peroxidase- (HRP-) conjugated secondary antibodies for 1 hr at room temperature, and then counterstained with hematoxylin. Finally, the sections were mounted and examined using a microscopy. Data were quantified by a blinded investigator. Nerve fiber densities were evaluated with ImagePro Plus 5.0 (Media Cybernetics, Bethesda, MD) as we previously reported [26]. Capillaries were defined as vessels with an internal diameter of <20 mm, and arterioles were defined as vessels with a diameter ranging from 20–100 mm. Capillary and arteriolar densities were measured as previously described [27]. Briefly, 10 random microscopic regions at the infarcted border were selected, and the vessels within the 100× microscopic field of each region were counted. The average vessel number in one section was used to assess vascular density.

In addition, paraffin-embedded samples from the apex, mid-LV, and base were sectioned (5-µm) and stained with Masson’s trichrome stain as described previously [28]. Fibrosis and the total LV area of each image were measured using Image-Pro Plus 5.0, and the infarct size percentage was calculated as fibrosis area/total LV area × 100.

### Western Blot (WB) Analysis

Expressions of NGF, vascular endothelial growth factor (VEGF) and glyceraldehyde-3-phosphate dehydrogenase (GAPDH) were detected by WB analysis. Proteins were extracted using a Nuclear and Cytoplasmic Protein Extraction Kit (Beyotime, Haimen, China). Protein concentration was quantified with a bicinchoninic acid (BCA) protein assay kit (Beyotime). From each extract, 80 µg total protein was separated with 10% SDS-PAGE and then transferred onto polyvinylidene fluoride (PVDF) membranes (Bio-Rad, Hercules, CA, USA). Membranes were blocked in 5% nonfat milk at room temperature for 1 hr and incubated overnight at 4°C with rabbit anti-VEGF (VEGF; 1∶1,000, Santa Cruz, CA, USA), anti-NGF (1∶1500, Epitomics, Burlingame, CA), or anti-GAPDH antibody (1∶3,000; CoWin Bioscience, Beijing, China). The next day, blots were incubated with appropriate horseradish peroxidase (HRP)-conjugated goat anti-rabbit IgG secondary antibody (1∶10,000, ZSGB-BIO, Beijing, China). Blots were developed using an enhanced chemiluminescence (ECL) detection kit (Millipore) and visualized using a FluroChem E Imager (ProteinSimple, Santa Clara, CA, USA). Then, the protein expression levels were quantified with Quantity AlphaEaseFCTM (Alpha Innotech, San Leandro, CA, USA) imaging software. Relative expression of target proteins was normalized to GAPDH.

### Real-time Quantitative Reverse Transcription Polymerase Chain Reaction (Real-time Quantitative RT-PCR)

Total RNA was isolated from myocardial samples with TRIzol reagent (Invitrogen, Carlsbad, CA, USA). Candidate gene expression was measured by real-time quantitative RT-PCR using a PrimeScript RT reagent kit (TaKaRa, Dalian, China) in a Mastercycler EP realplex detection system (Roche, Indianapolis, IN). For each sample, both GAPDH and target gene were amplified in duplicate in separate tubes. Each measurement was performed in triplicate. Gene expression was analyzed by the 2^-ΔΔCT^ method described by Livak and Schmittgen [29]. Primers for each gene used in this study were as follows:

NGF (forward 5′-tcgctcactccactatccacta-3′,

reverse 5′-gactcaacagggcaagcatac-3′);

TH (forward 5′-ggcttctctgaccaggtgtatc-3′,

reverse 5′-tagcaatctcttccgctgtgta-3′);

GAP-43 (forward 5′-gagggagatggctctgctact-3′,

reverse 5′-cttgtttaggctcctccttgg-3′);

VEGF (forward 5′-TAAATCCTGGAGCGTTCACTg-3′,

reverse 5′-tcacatctgcaagtacgttcg-3′);

and GAPDH (forward 5′-ACAGCAACAGGGTGGTGGAC-3′,

reverse 5′-tttgagggtgcagcgaactt-3′).

### Statistical Analyses

All data are expressed as mean ± standard deviation (SD). Unpaired t-tests were applied to compare values between two groups. Analysis of variance (ANOVA) was used to compare more than two groups followed by Tukey's test. Analyses were performed using the SPSS 17.0 software (SPSS Inc., Chicago, IL, USA). Values with P<0.05 were considered statistically significant.

## Results

### Intramyocardial Injection of NGF siRNA Persistently Suppressed NGF Expression in Post-infarcted Hearts

**Figure 1:**
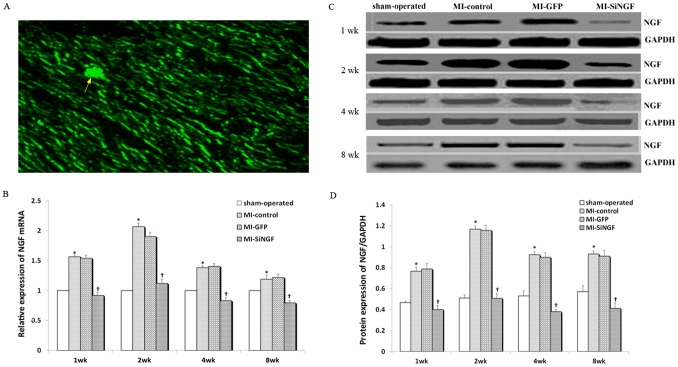
Transduction efficiency after intramyocardial injection of NGF siRNA *in vivo*. (A) Representative site of intramyocardial injection (arrowed) and GFP expression in infarcted hearts of rats in the MI-SiNGF group at 1 wk time point. (B) Relative expressions of NGF mRNA, detected by real-time quantitative RT-PCR, in the sham-operated, MI-control, MI-GFP and MI-SiNGF groups at various time points (1, 2, 4 and 8 wk) after intramyocardial injection of NGF siRNA. Relative gene expressions of NGF were analyzed by the 2^-ΔΔCT^ method taking those in the sham-operated groups as 1. (C) Expressions of NGF (27 KDa) and GAPDH (36 KDa), analyzed by Western blot, in the sham-operated, MI-control, MI-GFP and MI-SiNGF groups at various time points (1, 2, 4 and 8 wk) after intramyocardial injection of NGF siRNA. (D) Relative protein expressions of NGF. The protein expression levels in (C) were quantified with Quantity AlphaEaseFCTM imaging software. Relative expression of NGF was normalized to GAPDH. Data were presented as mean ± SD. *p<0.05 MI-control group vs. sham-operated groups, †*p<0.05* MI-SiNGF group vs. MI-control or MI-GFP groups. The results showed that expression levels of NGF mRNA and protein were induced in the MI-control group compared to the sham-operated group. NGF mRNA and protein in the MI-GFP group had no significant levels compared to the MI-control group, while those in the MI-SiNGF group were reduced compared to the MI-GFP group.

Transduction efficiency was initially assessed (Fig. 1A) by detecting the expression of the marker gene, GFP at 1 wk after intramyocardial injection of NGF siRNA in infarcted hearts of rats in the MI-SiNGF group. Rats were sacrificed to analyze NGF mRNA and protein expressions (Fig. 1B–D) at the infarcted border 1, 2 and 4 wk after injection (n = 3 per group). NGF expression was also detected at the end of the study (8 wk after injection, n = 8–10 per group). Both NGF mRNA and protein expression levels were significantly higher at various time points in the MI-control group compared to the sham-operated group (Fig. 1B and 1D, *p<0.01*), indicating that MI model was successfully established. No significant differences between MI-control and MI-GFP groups at the same time points were detected. The expression levels of NGF mRNA and protein were clearly lower in the MI-SiNGF group compared to the MI-GFP group at all tested time points (Fig. 1B and 1D, *p<0.01*). These data suggested that transduction was successfully performed, and NGF siRNA effectively inhibited the expression of NGF in infarcted hearts of rats in the MI-SiNGF group. However, NGF mRNA expressions in liver, kidney, spleen, and brain were similar among all groups.

### Lentiviral siRNA-mediated Silencing of NGF Aggravated MI-induced LV Dysfunction

Both echocardiographic and hemodynamic analyses were used to assess the influence of NGF siRNA on LV function at 8 wk time point after transduction (Table 1).

**Table 1:** Echocardiographic and Hemodynamic data at 8 wk after transduction.

	Sham-operated (n = 8)	MI-control (n = 9)	MI-GFP (n = 9)	MI-SiNGF (n = 10)
LVIDd (mm)	6.23±0.31	7.37±0.42*	7.31±0.47	8.04±0.54†
LVIDs (mm)	3.46±0.27	5.72±0.36*	5.99±0.42	6.97±0.47†
FS (%)	43.3±4.6	19.0±2.0*	18.4±2.3	13.4±1.9†
EF (%)	76.2±5.2	42.3±3.0*	45.27±4.0	29.1±2.3†
HR (beats/min)	413.3±24.1	435.2±19.7*	429.5±23.2	467±14.2†
MAP (mmHg)	114.2±6.7	91.5±7.1*	94.2±8.9	84.3±7.4†
LVEDP (mmHg)	3.1±1.0	6.4±1.5*	5.9±1.8	11.2±2.0†
LVESP (mmHg)	121.2±7.7	91.2±5.9*	87.3±6.7	83.4±5.1†
dP/dt_max_ (mmHg/s)	5611.2±107	3921.3±86.2*	3869.51±90.2	2407.8±76.3†
dP/dt_min_ (mmHg/s)	−4063.5±134	−2473.5±79.4*	−2503.7±86.2	−1937.5±90.4†
EDV (µl)	312.1±16.2	483.2±33.7*	467.6±41.4	671.7±57.6†
ESV (µl)	102.7±10.7	293.4±19.4*	287.2±23.1	463.2±47.4†

Values were means±SD. LVIDd, left ventricular internal diameter at end-diastolic phase; LVIDs, left ventricular internal diameter at end-systolic phase; EF: left ventricular ejection fraction; FS: left ventricular fractional shortening; HR, heart rate; MAP: mean arterial pressure; LVEDP: LV end-diastolic pressure; LVESP: LV end-systolic pressure; dP/dt_max_: the maximal rates of increase in left ventricular pressure; dP/dt_min_: the maximal rates of decrease in left ventricular pressure.

* *P<0.05* vs. Sham-operated group;

† *P<0.05* vs. MI-GFP group.

The LVIDd and LVIDs were higher, and the LV fractional shortening (FS) and ejection fraction (EF) were lower in the MI-control group compared with those in the sham-operated group (*p*<0.05). Rats in the MI-GFP group showed similar levels of LVIDd, LVIDs, FS, and EF as rats in the MI-control group (*p*>0.05). Compared to the MI-GFP group, the LVIDd and LVIDs in the MI-SiNGF group were higher, and FS and EF were lower (*p*<0.05).

In the MI-control group, HR was higher, while MAP was lower than that in the sham-operated group (*p*<0.05). No significant differences in the levels of HR and MAP were detected between the MI-control and MI-GFP groups (*p*>0.05). However, compared to the MI-GFP group, rats in the MI-SiNGF group had a higher level of HR and a lower level of MAP (*p*<0.05).

Compared to the sham-operated group, LVESP and dP/dt_max_ were significantly lower in the MI-control group (*p*<0.05). No significant differences in the levels of LVESP and dP/dt_max_ were observed between the MI-control and MI-GFP groups (*p*>0.05). Both levels were lower in the MI-SiNGF group compared to the MI-GFP group (*p*<0.05). Conversely, higher levels of LVEDP, dP/dt_min_, ESV and EDV were measured in the MI-control and MI-SiNGF groups when compared to the sham-operated and MI-GFP group, respectively (*p*<0.05).

### Effect of NGF Silencing on the Infarcted Area

**Figure 2:**
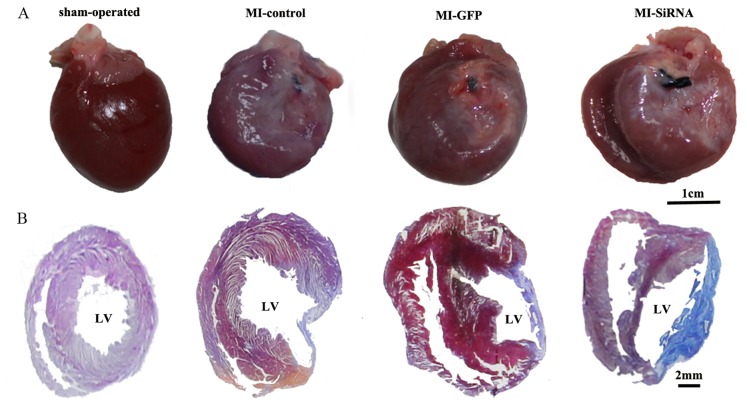
Representative images for infarcted hearts at 8 wk time point after MI. (A) Macroscopical view of the infarcted heart. (B) Masson’s trichrome staining of infarcted area.

Eight wk after infarction, a large infarcted area with a collapsed and pale left ventricular wall was seen under the ligated silk. Compared to the MI-GFP group, larger infarcted area was detected accompanying global enlargement of the heart in the MI-SiRNA group (Fig. 2A). Masson’s trichrome staining revealed viable and fibrous tissue in the heart samples (Fig. 2B). Infarcted myocardium, which was replaced with fibroblasts and collagen, appeared blue, and viable myocardium appeared red. The infarcted area was larger in the MI-SiNGF group at 8 wk, compared to the MI-GFP group (63.4±9.2% vs. 43.7±5.2%, *p<0.01*).

### NGF Silencing Attenuated Sympathetic Nerve Sprouting after MI

**Figure 3:**
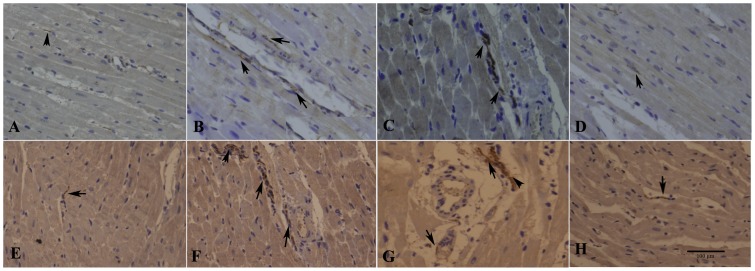
Histological study of cardiac nerve fibers at infarct border in sham-operated and infarcted hearts. (A–D) GAP-43-positive nerve fibers (arrows) in sham-operated, MI-control, MI-GFP, and MI-SiRNA groups, respectively. Few GAP43-positive nerve fibers were observed in the sham-operated and MI-SiRNA groups. (E–H) TH-positive nerve fibers (arrows) in sham-operated, MI-control, MI-GFP, and MI-SiRNA groups, respectively. In the MI-GFP and MI-control groups, nerve fibers were disordered or unevenly distributed. Conversely, relatively normal nerve fibers were observed in the MI-SiRNA group. Bar = 100 µm.

Compared to the sham-operated group, GAP-43 and TH mRNA levels in the MI-control group were significantly increased at the infarcted border (∼2 mm around the infarcted area) and in the non-infarcted LV (>2 cm away from the infarcted scar) at 8 wk time point after MI (*p<0.01* and *p<0.05* respectively, Table 2). Rats in the MI-GFP and MI-control groups showed non-significant levels (*p>0.05*). However, GAP-43 and TH mRNA expressions at both sites were lower in the MI-SiNGF group (*p<0.01* vs. the MI-GFP group, Table 2). Immunohistochemical staining revealed that both GAP-43- and TH-positive nerve densities were significantly higher in the MI-control group compared to the sham-operated group (Fig. 3A, 3B, 3E, 3F, Table 2). Rats in the MI-SiNGF group had less TH-immunoreactive nerve density at two corresponding sites compared to MI-GFP rats (Both *p<0.01* respectively, Fig. 3G, 3H, Table 2). Similar to the result observed for TH, the GAP-43-positive nerve density was significantly lower in the MI-SiNGF group compared with that in the MI-GFP group (*p<0.01* at both the infarct bordered and non-infarcted LV, (Fig. 3C, 3D, Table 2).

**Table 2:** Histological and RT-PCR results of TH- and GAP 43 expression.

	mRNA expression				Nerve fiber density (*um^2^/mm^2^*)			
	GAP 43		TH		GAP 43		TH	
	infarcted border	non-infarcted LV	infarcted border	non-infarcted LV	infarcted border	non-infarcted LV	infarcted border	non-infarcted LV
**sham-operated**	1	1	1	1	609±73	548±62	1318±134	1339±164
**MI-control**	2.97±0.31*	2.17±0.08*	3.38±0.38*	2.35-±0.14*	1407±169*	1072±73*	3458±440*	2591±232*
**MI-GFP**	3.03±0.29	2.15±0.08	3.35±0.19	2.43±0.16	1404±109	1082±94	3692±350‡	2580±258
**MI-SiNGF**	0.86±0.05†	0.88±0.04†	1.18±0.80†	1.12±0.04†	596±64†	580±44†	1295±167†	1449±99†

Data were expressed as means ± SD.

* *P<0.05* vs. Sham-operated group;

† *P<0.05* vs. MI-GFP group.

### NGF Silencing Suppressed Angiogenesis and Decreased Microvessel Density

**Figure 4:**
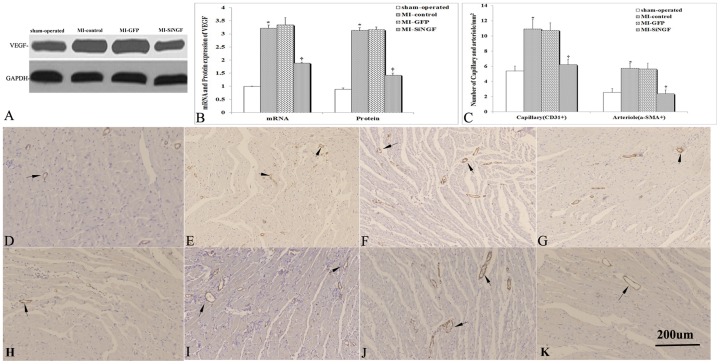
VEGF expression, CD31- and α-SMA-positive vessels at infarct borders 8 wk after NGF silencing. (A) Representative Western blot autoradiograms of VEGF (46 KDa) and GAPDH proteins. (B) MI increased the expression levels of VEGF mRNA and protein. NGF silencing reduced the expression levels of VEGF mRNA and protein. (C) Quantification of capillaries and arterioles at the infarct border 8 wk after MI. (D–G) Immunohistochemical staining of capillaries with an antibody against CD31 in sham-operated, MI-control, MI-GFP, and MI-SiRNA groups, respectively. (H–K) representative images of arterioles with an antibody against α-SMA in sham-operated, MI-control, MI-GFP, and MI-SiRNA groups, respectively. Data are mean ± SD (n = 8–10 per group). *p<0.05 vs. sham-operated, **p>0.05 vs. MI-control, and †*p<0.05* vs. MI-GFP. Bar = 200 µm.

Compared to the sham-operated group, VEGF was up-regulated at the infarcted border 8 wk after MI in the MI-control group as assessed by WB (Fig. 4A and 4B) and real-time quantitative RT-PCR (Fig. 4B). VEGF mRNA and protein expression levels in the MI-control and MI-GFP groups were not significantly different (*p>0.05*, Fig. 4B). MI induced VEGF was inhibited by lentiviral NGF siRNA transduction (*p<0.01* vs. the MI-GFP group, Fig. 4B).

Capillary and arteriolar densities were histologically analyzed using CD31- and α-SMA positive vessels at the infarcted border (Fig. 4D-4G and Fig. 4H–4K, respectively). Arteriolar density was higher at the infarcted border in the MI-control group compared to the sham-operated group (Fig. 4D and 4E). However, the MI-SiNGF group had significantly lower arteriolar density (2.3±0.5/mm^2^ vs. 5.6±0.8/mm^2^, *p<0.01*, Fig. 4F and 4H) compared to the MI-GFP group. Similar results were observed with respect to capillary density. The MI-SiNGF group had significantly less capillary in contrast to MI-GFP rats (6.2±0.7/mm^2^ vs. 10.7±1.1/mm^2^, *p<0.05*, Fig. 4J–4K).

## Discussion

Cardiac NGF levels are dramatically increased following MI. In infarcted hearts, elevation of NGF expression may lead to sympathetic nerve sprouting which potentially contributes to the later genesis of arrhythmias but may also favor the healing process [6]. Further elucidation of NGF effects may increase our understanding of post-infarct remodeling processes. In the present study, targeted intra-cardiac administration of NGF siRNA was used *in vivo* to knock down endogenous NGF in infarcted rat hearts. Our data revealed that targeted intra-cardiac administration of NGF siRNA reduced nerve sprouting and decreased sympathetic nerve density. Specifically, NGF silencing was associated with the increase in infarct size, reductions in capillary and arteriolar densities, and the enhancement of cardiac dysfunction in a rat MI model. Our results indicated that NGF is not an ideal drug candidate despite its therapeutic potential.

Consistent with the previous study [9], we found that, following MI, there was a dynamic expression pattern of NGF protein and mRNA, which increased from 1 to 2 wk, and then gradually decreased at 4 and 8 wk time points compared to the sham-operated group. At 8 wk time point after MI, heterogeneous sympathetic nerve sprouting (GAP-43-positive nerve fibers) and sympathetic innervation (TH-positive nerve fibers) were observed accompanying NGF elevation. However, injection of the well-packaged and validated NGF lentiviral si-RNA continuously downregulated NGF in the in vivo heart [8]. Lentiviral vectors can stably integrate into the host genome and facilitate long-term transgene expression with high transfection efficiency, which are thought to be one of the most promising vehicles for the efficient delivery of genetic information in basic research and gene therapy approaches [30]. Our data showed that NGF was continuously down-regulated in vivo in infarcted hearts by NGF silencing. NGF expression was correlated with sympathetic nerve fiber density in target tissues. NGF silencing clearly decreased GAP-43-positive and TH-positive nerve fibers density. These findings indicated that NGF silencing could inhibit sympathetic nerve sprouting and innervation.

Furthermore, lentiviral-mediated NGF siRNA decreased angiogenesis at both capillary and arteriolar levels and reduced VEGF expression. Specifically, we observed that targeted NGF silencing could enlarge infarct size and aggravate cardiac dysfunction by reducing EF, FS, LVESP, and dp/dtmax and elevating EDV, SDV, LVEDP, and dp/dtmin. Angiogenesis is a critical physiological phenomenon for cardiac repair, and impaired angiogenesis may cause cardiac rupture or immature scar tissue formation [31]. Spontaneous neovascularization after MI accounts for improved perfusion and delivery of oxygen and nutrients to salvage the surviving myocardium [32]. In addition, cardiomyocyte growth, survival, and contractile function depend upon microvascular function and angiogenesis [32]. NGF directly induces the formation of capillaries to accelerate blood flow recovery in ischemic muscle [33]. Hence, we speculated that reduced VEGF production and angiogenesis by NGF silencing contribute to the increased infarct size and subsequent cardiac dysfunction. Another potential mechanism is involved in the deficiency of direct protection of NGF as an autocrine prosurvival factor for cardiomyocytes [33]. NGF gene transfer supports cardiomyocytes survival in post-infarcted rats [19]. And NGF was directly anti-apoptotic in cardiomyocytes and promoted their survival in infarcted hearts [20]. Thus, up-regulation of NGF may participate in cardiac repair in post-infarcted hearts and NGF decline may be negative, promoting anatomical remodeling and resulting in more severe cardiac dysfunction.

Previous studies have showed that dexamethasone [14], nicorandil [15], and metoprolol [13] can improve sympathetic nerve remodeling partly through down-regulating NGF. Infarct size is not significantly affected in resveratrol-treated infarcted rats which have a decreased NGF level [16]. However, application of these medicines may bring cardiac protective effects and improve post-MI anatomical remodeling via multiple ways including their anti-oxidant and anti-inflammatory effects [16]. Besides, differently reduced level of NGF may be another factor which influences its effect. Hence, further characterization of NGF changes at different stages may improve our understanding of complete information about the time course of the NGF and its contribution to damage expression/repair.

In conclusion, we have identified NGF as an important factor in pathophysiologic process of remodeling in post-infarcted hearts. Direct local reduction of NGF synthesis results in decreased activity of sympathetic nerve sprouting, attenuated angiogenesis, augmented infarct size and aggravated cardiac dysfunction. Properly controlling the expression of NGF after MI via some medicines may bring pleiotropic benefits.
